# Histone Deacetylase 9 Gene Deletion Ameliorates Aging‐Related Adipose Tissue Senescence and Mitochondrial Dysfunction in Mice

**DOI:** 10.1111/acel.70519

**Published:** 2026-04-25

**Authors:** Brandee Goo, Samah Ahmadieh, Praneet Veerapaneni, Hong Shi, David S. Kim, Mourad Ogbi, Ronnie Chouhaita, Nicole Cyriac, Stephen Cave, Lingling Liu, Yong Zhang, Quansheng Du, Ha Won Kim, Yun Lei, Xin‐Yun Lu, Neal L. Weintraub

**Affiliations:** ^1^ Vascular Biology Center Medical College of Georgia at Augusta University Augusta Georgia USA; ^2^ Department of Medicine Medical College of Georgia at Augusta University Augusta Georgia USA; ^3^ Neuroscience and Regenerative Medicine Medical College of Georgia at Augusta University Augusta Georgia USA

**Keywords:** adipose tissue, HDAC9, mitochondria, senescence, thiosulfate sulfurtransferase

## Abstract

Cellular senescence and mitochondrial dysfunction are prevalent in adipose tissues and disrupt metabolic homeostasis during aging, but the mechanisms are poorly understood. Here, we investigated the role of histone deacetylase 9 (HDAC9), an epigenetic regulator of adipogenic differentiation, in aging‐related adipose tissue senescence and mitochondrial dysfunction. HDAC9 expression correlated positively with age in mouse adipose tissues. Compared to age‐matched wild‐type (WT) mice, *Hdac9* knockout (KO) mice gained less weight and had reduced fat mass during aging, in conjunction with reduced senescence‐associated beta‐galactosidase (SABG) staining and expression of senescence markers in adipose tissues. Additionally, preadipocytes isolated from *Hdac9* KO mice exhibited reduced baseline and stress‐induced senescence compared to WT mice. Mechanistically, HDAC9 gene deletion resulted in coordinated upregulation of mitochondria‐associated genes, in association with increased mitochondrial DNA content and adipose tissue mitochondrial oxygen consumption parameters (e.g., increased basal respiration, proton leak). Furthermore, thiosulfate sulfurtransferase (TST), whose downregulation is associated with mitochondrial dysfunction, was reduced in adipose tissues of aging mice and upregulated by HDAC9 gene deletion. Finally, silencing TST in preadipocytes upregulated expression of senescence markers and increased SABG staining. We conclude that deletion of HDAC9 ameliorates the development of adipose tissue senescence and mitochondrial dysfunction with aging, at least in part via upregulation of TST, suggesting that targeting HDAC9 may be a promising strategy to maintain healthy adipose tissue during aging.

## Graphical abstract

Cellular senescence and mitochondrial dysfunction disrupt adipose tissue homeostasis during aging, but mechanisms remain unclear. HDAC9 expression increases with age in mouse adipose tissue and Hdac9 knockout mice showed reduced fat mass, senescence markers, and enhanced mitochondrial gene expression, respiration, and thiosulfate sulfurtransferase (Tst) expression. Tst transcription is regulated by HDAC9, and silencing of Tst in preadipocytes increased senescence markers expression, implicating HDAC9‐Tst signaling in adipose tissue aging.

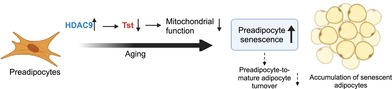

## 1 Introduction

Adipose tissue undergoes metabolic and structural changes during aging, including cellular senescence, immune cell infiltration, impaired lipid storage and mitochondrial dysfunction (Palmer and Jensen 2022). Aged adipose tissue is a major source of chronic, low‐grade sterile inflammation, and it can have a profound effect on systemic levels of inflammation via the senescence‐associated secretory phenotype (SASP) (Chaib et al. 2021; Conley et al. 2020; Tchkonia et al. 2010). Senescence of preadipocytes, precursor cells that reside in adipose tissues and are capable of differentiating into mature adipocytes, reduces the capacity to replenish dead adipocytes, leading to hypertrophy of existing adipocytes, worsening adipose tissue inflammation and ectopic lipid deposition in other organs (Caso et al. 2013; Chatterjee, Basford, Knoll, et al. 2014; Chatterjee, Basford, Yiew, et al. 2014; Kuk et al. 2009; Onate et al. 2012; Rawshani et al. 2020). Interestingly, reducing senescent cell burden by senolytic, senomorphic, or genetic manipulations in aging models has been shown to improve adipose tissue function, including insulin sensitivity, glucose tolerance and adipogenesis (Palmer et al. 2019; Wang et al. 2022; Xu, Palmer, et al. 2015; Xu, Tchkonia, et al. 2015).

Similar to the state of obesity, aging is associated with redistribution of adipose tissue from the subcutaneous to the visceral compartment (Mancuso and Bouchard 2019; Palmer and Kirkland 2016). Compared to subcutaneous fat, visceral fat is associated with higher levels of inflammation and cellular senescence, impaired adipogenic differentiation, and reduced mitochondrial function, a hallmark of aging that is closely intertwined with cellular senescence (Lopez‐Otin et al. 2013). However, despite the important role of adipose tissues in systemic aging, the mechanisms of adipose tissue aging are poorly understood.

Histone deacetylase 9 (HDAC9) is a class IIa HDAC that has both epigenetic and non‐epigenetic functions. We previously showed that HDAC9 is a negative regulator of adipogenic differentiation, and its expression is rapidly downregulated in preadipocytes in response to adipogenesis‐inducing stimuli, thereby enabling acquisition of the mature adipocyte phenotype (Chatterjee et al. 2011). In mice, global *Hdac9* gene deletion reduces weight gain during high fat diet (HFD) feeding through increased energy expenditure. *Hdac9* KO mice fed a HFD had reduced adipocyte hypertrophy and adipose tissue inflammation, and improved insulin sensitivity and glucose tolerance (Chatterjee, Basford, Knoll, et al. 2014; Chatterjee, Basford, Yiew, et al. 2014), demonstrating that HDAC9 plays a crucial role in adipogenesis and obesity‐associated metabolic disease. However, the role of HDAC9 in adipose tissue aging is unknown (Chatterjee, Basford, Yiew, et al. 2014) wn. In this study, we show that *Hdac9* expression is increased in adipose tissues during aging. Using *Hdac9* KO mice at various ages, we investigated the role of HDAC9 in aging of adipose tissues. We show that deletion of HDAC9 reduces accumulation of senescent cells in aging adipose tissue in vivo and protects preadipocytes from becoming senescent in vitro. Furthermore, adipose tissues from *Hdac9* KO mice exhibited improved mitochondrial function compared to those from WT mice. Moreover, we identified upregulation of thiosulfate sulfurtransferase (TST) as a potential underlying mechanism whereby deletion of *Hdac9* improves mitochondrial function and reduces cellular senescence.

## 2 Materials and Methods

### 2.1 Ethics Statement

The work was conducted in accordance with ARRIVE guidelines. Animal experimental protocols were approved by the Institutional Animal Care and Use Committee at the Medical College of Georgia at Augusta University (protocol approval #: 2013‐0528) and complied with National Institute of Health guidelines. All experiments were carried out in accordance with institutional biosafety and chemical safety guidelines and regulations.

### 2.2 Mice

C57BL/6 mice were purchased from Jackson Laboratories and *Hcac9* KO mice were obtained from Dr. Eric Olson (Zhang et al. 2002), and backcrossed with C57BL/6J in our laboratory. Heterozygous (+/−) mice were crossed to obtain *Hdac9* KO (−/−) and wild‐type (+/+) littermate controls for our experiments. All mice were male unless otherwise specified. Mice were housed at thermoneutral temperature (27.5°C–30°C) and maintained on chow diet (CD, Harlan Teklad, LM‐485). All animal study protocols were approved by the Institutional Animal Care and Use Committee of Augusta University following appropriate guidelines.

### 2.3 Glucose Tolerance Test (GTT) and Insulin Tolerance Test (ITT)

For GTT, mice were fasted for 6 h. Blood was collected from the tail vein immediately prior to and 15, 30, 60, 90, and 120 min following intraperitoneal (IP) injection of glucose (Sigma) at 2 g/kg body weight. For ITT, mice were fasted for 6 h. Blood was collected from the tail vein immediately prior to and 15, 30, 60, and 90 min following IP injection of 0.75 units regular insulin/kg body weight (Humulin).

### 2.4 Whole‐Body Nuclear Magnetic Resonance (NMR)

Fat mass and lean mass were measured in approximately 1‐year‐old mice using NMR spectroscopy (Bruker Minispec LF90II) as previously reported (Chatterjee, Basford, Knoll, et al. 2014; Chatterjee, Basford, Yiew, et al. 2014).

### 2.5 Adipose Tissue Fractionation and Preadipocyte Culture

Murine adipose tissues were separated into the stromovascular fraction (SVF) and mature adipocyte fraction (MAF) by collagenase digestion as previously described (Yiew et al. 2019). Primary preadipocytes were cultured from the SVF in preadipocyte growth medium (Cell Applications) as previously described (Chatterjee et al. 2011). To induce senescence, cells were plated into a 6‐well dish and treated with 30 μM of hydrogen peroxide (H_2_O_2_) at Days 1, 4, and 7. Then, cells were collected or fixed on Day 10 for further analysis. In separate experiments, cells were plated into 35 mm dishes and exposed to 2 s of ultraviolet light 20 in. away from the light source in the cell culture hood. Cells were then washed with PBS and cultured for 5 days before collection or fixation for further analysis.

### 2.6 Senescence‐Associated β‐Galactosidase Staining (SABG)

Whole adipose tissue, MAF, and cultured preadipocytes were stained for SABG using the senescence β‐galactosidase staining kit (CST #9860) per manufacturer's instructions. Briefly, samples were washed with phosphate buffered saline (PBS) and fixed for 10 min for MAF and preadipocytes, or 30 min for approximately 100 mg whole adipose tissue pieces. Samples were washed three times to remove fixative and incubated in staining solution for 12 h.

### 2.7 Quantitative PCR

Total RNA was extracted from tissues and cells with Qiazol and processed with the RNeasy Lipid Tissue Mini Kit (Qiagen). RT‐PCR quantification of mRNA levels was performed using SYBR green qRT‐PCR kits (Agilent Technologies, abm). Fold change was calculated by ΔΔCt method using normalization to *Arbp* for mouse samples and *Gapdh* for human samples. Total DNA was extracted from adipose tissues with the DNeasy Kit (Qiagen). RT‐PCR quantification of mitochondrial DNA was performed with SYBR green qRT‐PCR kit (abm). Relative mitochondrial DNA copy number was determined by the ratio of mitochondrial CoxII gene to nuclear β‐globin gene. Primer sequences are shown in Table S1.

### 2.8 RNA Sequencing and Analysis

RNA sequencing was performed on RNA from VF from 10‐month‐old male *Hdac9* KO and littermate WT mice by GeneWiz. GSEA and Network Topology‐based analysis were performed with WEB‐based GEne SeT AnaLysis Toolkit 2017 (WebGestalt2017; http://www.webgestalt.org). GSEA was performed using the clusterProfiler R package with pre‐ranked gene lists. Genes were ranked by the signed product of log_2_(fold change) and −log_10_(adjusted *p*‐value) from differential expression analysis. Gene sets from GO Biological Process and KEGG pathways (MSigDB) were tested with the following parameters: minimum gene set size of 15 genes, maximum gene set size of 500 genes, and 1000 permutations for significance testing. Pathways with FDR < 0.25 were considered significantly enriched. For visualization, the top 20 pathways ranked by FDR are displayed in dot plots, where dot size represents gene set size and color represents FDR *q*‐value.

### 2.9 Western Blot

Protein was extracted from adipose tissue in RIPA buffer with protease inhibitors with a Tissue‐Tearer (BioSpec), followed by centrifugation and separation by SDS‐PAGE. Proteins were transferred to nitrocellulose membrane, probed with appropriate antibody and developed with ECL. Antibodies were obtained from Biorbyt (HDAC9; 214926), BD Transduction (HSP90; 610419), Invitrogen (GAPDH; AM4300), CST (P16; 68410, P21; 37543), and GeneTex (TST; GTX114858).

### 2.10 Seahorse MitoStress Assay of Adipose Tissue Explants

Seahorse MitoStress assay was performed on 4–6 mg adipose tissue explants using the Seahorse XFe24 Islet Capture Microplate on the Seahorse XFe24 Analyzer (Agilent). Adipose tissue samples were carefully standardized for size and thickness to minimize oxygen diffusion limitations. Explants were evenly distributed to avoid stacking or uneven exposure to assay media. FCCP concentration was titrated to identify the dose producing maximal respiratory stimulation without inducing non‐specific toxicity. The final concentrations of mitochondrial inhibitors were 10 μM oligomycin (Sigma), 8 μM FCCP (Sigma), 20 μM rotenone (Sigma) and 16 μM antimycin A (Sigma) in Seahorse XF Dulbecco's Modified Eagle Medium (DMEM) (Agilent) supplemented with 25 mM glucose (Agilent), 1 mM pyruvate (Agilent) and 2 mM glutamine (Agilent).

### 2.11 siRNA Transfection

Mouse primary preadipocytes were plated into a 6‐well plate and starved with DMEM (gibco #1185‐084) overnight. At 50% cell confluency, preadipocytes were transfected with Rhodanese siRNA (Santa Cruz Biotechnology #sc‐36419) or control siRNA‐A (Santa Cruz Biotechnology #sc‐37007) using the Lipofectamine 3000 transfection kit (Invitrogen #L3000001) in Opti‐MEM (gibco #31985‐070) for 24 h according to the manufacturer's protocol. Non‐siRNA (mock transfection) groups were used as controls. Then, cells were further incubated in preadipocyte growth media for 24 h and harvested for further analysis.

### 2.12 Chromatin Immunoprecipitation (ChIP) Assay

ChIP assays were performed using a ChIP assay kit (Epigentek) according to the manufacturer's instructions and as previously described (Chatterjee et al. 2011). Briefly, preadipocytes were cross‐linked with 1% formaldehyde for 10 min at room temperature and then lysed using the lysis buffer included in the kit. Chromatin was sheared by sonication. After centrifugation, the supernatants containing protein‐chromatin complexes were immunoprecipitated with ChIP‐grade antibodies against IgG, H3K4ac (Abcam), H3K9ac (Abcam), and H3K18ac (Abcam). Protein‐DNA complexes were pulled down and eluted using the buffer solution included in the kit. Purified DNA was quantified by real‐time PCR. The following primers were used to detect enrichment of DNA fragments in the predicted Tst promoter region (forward: 5′‐GGAGCCCGGATATAGTAGGACTAGA‐3′, reverse: 5′‐TTCGTCAGGAAGTCCATGAA‐3′).

### 2.13 Statistical Analysis

GraphPad Prism version 9 software (GraphPad Software Inc.) was used for statistical calculations. Data are expressed as mean ± SEM (standard error of the mean). The Shapiro–Wilk test was used to assess the normality. The *F* test was used to assess equality of variance between groups. An unpaired two‐tailed *t*‐test was used to compare between two groups with equal variances. Welch's correction was used for two‐sample comparison between groups with unequal variances. A two‐way analysis of variance (ANOVA) followed by Tukey's post hoc tests was used for comparisons between multiple groups. Simple linear regression was used for correlation analysis. *p*‐value of < 0.05 was considered significant.

## 3 Results

### 3.1 Aging Is Associated With Increased Expression of HDAC9 in Mouse Adipose Tissues

**FIGURE 1:**
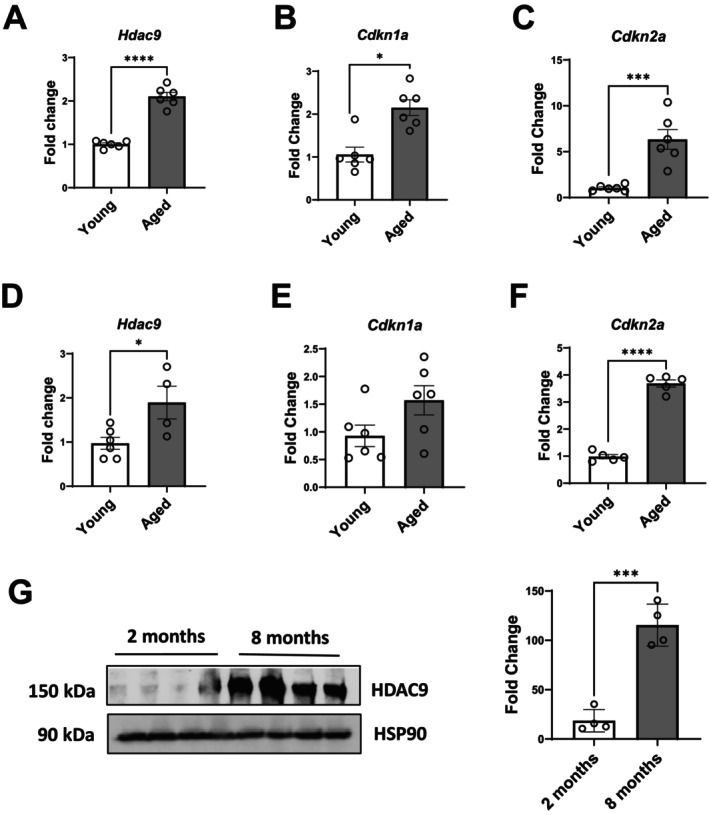
HDAC9 expression is upregulated in adipose tissues of aged mice and humans. qRT‐PCR analysis of *Hdac9* (A, D), *Cdkn1a* (B, E) and *Cdkn2a* (C, F) from VF (A–C) and SQ (D–F) adipose tissue from young (2–3‐month‐old) and aged (14–20‐month‐old) WT male mice as evaluated by qRT‐PCR (*n* = 4–6). (G) HDAC9 protein expression in VF from 2‐month‐old versus 8‐month‐old WT male mice as evaluated by western blot (*n* = 4). VF, visceral fat; SQ, subcutaneous fat. Data represent mean ± SEM. *p < 0.05, ***p < 0.001.

We previously showed that HFD‐induced obesity was associated with increased HDAC9 expression in adipose tissues (Chatterjee, Basford, Knoll, et al. 2014; Chatterjee, Basford, Yiew, et al. 2014). To investigate whether aging has a similar effect on adipose tissue HDAC9 expression, we compared gene expression between young (3‐month‐old) and aged (20‐month‐old) male WT mice. *Hdac9* mRNA expression was increased in visceral fat of aged mice (Figure 1A), in conjunction with elevated expression of senescence markers cyclin dependent kinase inhibitor 1A (*Cdkn1a* which encodes p21 protein) and *Cdkn2a* (which encodes p16 protein) in (Figure 1B,C). Similar results were found in subcutaneous fat (Figure 1D–F). In both subcutaneous and visceral fat of aging mice, *Hdac9* expression trended to be higher in the mature adipocyte fractions (MAF), while expression of *Cdkn1a* and *Cdkn2a* trended to be higher both in the MAF and the stromovascular (SV) fractions (Figure S1A–C). HDAC9 protein expression was also significantly increased in visceral fat of 8‐month old compared to 2‐month‐old mice (Figure 1G). These results demonstrate that HDAC9 expression is upregulated with aging in adipose tissues.

### 3.2 Effects of HDAC9 Gene Deletion on Metabolic Parameters in Aging Mice

**FIGURE 2:**
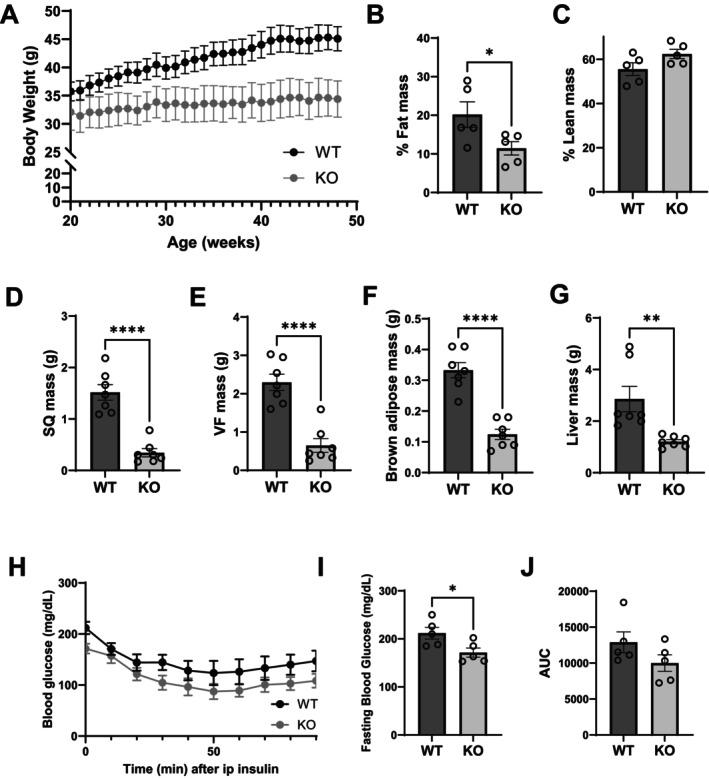
HDAC9 gene deletion protects against age‐associated metabolic alterations in mice. (A) Weekly body weight measurements of male WT and HDAC9 KO mice (*n* = 7/group). NMR measurement of fat (B, *n* = 5/group) and lean body composition (C, *n* = 5/group) and tissue weights (D: SQ, E: VF, F: Brown fat, G: Liver, *n* = 7/group) of 12‐month‐old mice. Insulin tolerance test (H), fasting glucose (I) and area under curve (AUC) analysis (J) of 12‐month‐old WT and HDAC9 KO mice (*n* = 5/group). VF, visceral fat; SQ, subcutaneous fat. Data represent mean ± SEM. *p < 0.05, **p < 0.01, ****p < 0.0001.

To assess the effects of HDAC9 gene deletion on metabolic parameters during aging, we first measured body weight of WT and *Hdac9* KO mice from 4 to 12 months of age. Consistent with our previous findings in young mice, aging *Hdac9* KO mice gained less weight compared to WT mice (Figure 2A). Nuclear magnetic resonance (NMR) evaluation of whole‐body composition showed selective reduction in fat mass in 12‐month‐old *Hdac9* KO mice (Figure 2B,C), along with reduced tissue weight of subcutaneous and visceral fat in *Hdac9* KO mice compared to WT mice (Figure 2D,E). Additionally, the interscapular brown adipose depot and the liver weighed less in 12‐month‐old male *Hdac9* KO mice compared to WT mice (Figure 2F,G). Female *Hdac9* KO mice showed a similar trend towards reduced body weight and adiposity during aging (Figure S2). Furthermore, 12‐month‐old male *Hdac9* KO mice had significantly lower fasting blood glucose levels, and a trend towards enhanced insulin sensitivity, compared to WT mice (Figure 2H–J).

### 3.3 HDAC9 Gene Deletion Protects Against the Development of Senescence in Adipose Tissues

**FIGURE 3:**
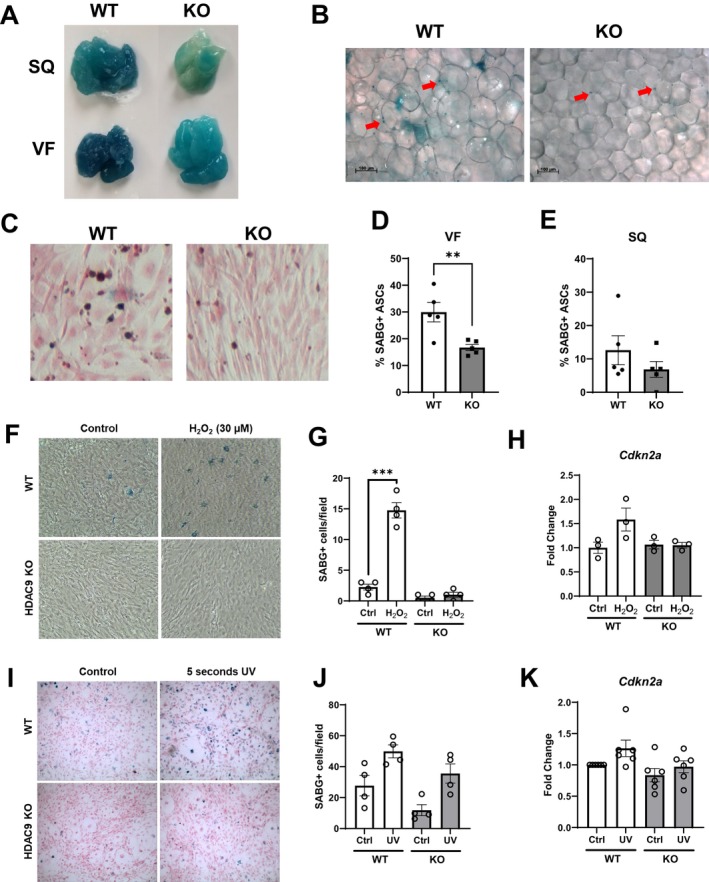
HDAC9 gene deletion protects against the development of adipose tissue senescence. Representative gross (A) and 7.5× images (B) of SABG‐stained whole adipose tissues of 12‐month‐old mice. Arrows indicate SABG positivity. (C) Representative images of untreated SABG‐stained primary preadipocytes from VF of 12‐month‐old mice. Quantification of SABG‐stained primary preadipocytes from VF (D) and SQ (E) from 12‐month‐old mice (*n* = 5/group). (F, G) Representative SABG images (F) and quantification (G) of primary preadipocytes isolated from VF and treated with 30 μM H_2_O_2_ (*n* = 4/group). (I, J) Representative SABG images (I) and quantification (J) of primary preadipocytes isolated from VF and treated with 5 s UV light (*n* = 4/group). Relative mRNA expression of senescence marker, *Cdkn2a*, in preadipocytes treated with H_2_O_2_ (H, *n* = 3/group) or UV light (K, *n* = 6/group). VF, visceral fat; SQ, subcutaneous fat. Data represent mean ± SEM. **p < 0.01, ***p < 0.001.

Whole adipose tissues (subcutaneous and visceral fat) from 12‐month‐old *Hdac9* KO mice exhibited significantly less SABG staining compared to WT mice (Figure 3A,B), suggesting that HDAC9 gene deletion reduces the senescent phenotype of cells within adipose tissues. We also observed less SABG staining in mature adipocytes isolated from visceral fat of *Hdac9* KO mice compared to WT mice (Figure S1D). Next, we performed SABG staining in preadipocytes isolated from adipose tissues of 12‐month‐old mice. Interestingly, a lower percentage of preadipocytes from visceral fat of *Hdac9* KO mice stained positively for SABG compared to those from WT mice (Figure 3C,D). Preadipocytes from subcutaneous fat also showed a trend towards decreased SABG (Figure 3E). These results suggest that HDAC9 gene deletion reduces senescence in adipose tissues of aging mice.

To determine whether HDAC9 promotes adipose senescence in a cell‐autonomous manner, preadipocytes isolated from visceral fat of WT and *Hdac9* KO mice were exposed in vitro to H_2_O_2_, a senescence‐inducing stressor. Notably, H_2_O_2_‐induced senescence, as assessed by SABG staining, was markedly reduced in preadipocytes isolated from *Hdac9* KO mice compared to WT mice (Figure 3F,G). In addition, the induction of *Cdkn2a* by H_2_O_2_ was abrogated by HDAC9 gene deletion (Figure 3H). We also briefly exposed preadipocytes to UV light, another known senescence inducer. SABG staining (Figure 3I,J) and *Cdkn2a* expression (Figure 3K) tended to be higher in preadipocytes from WT versus *Hdac9* KO mice. These results suggest that HDAC9 gene deletion protects preadipocytes from stress‐induced senescence and reduces the accumulation of senescent cells in adipose tissues of aging mice.

### 3.4 HDAC9 Gene Deletion Regulates Expression of Metabolic, Inflammatory, and Senescence Signaling Pathways in Visceral Fat

**FIGURE 4:**
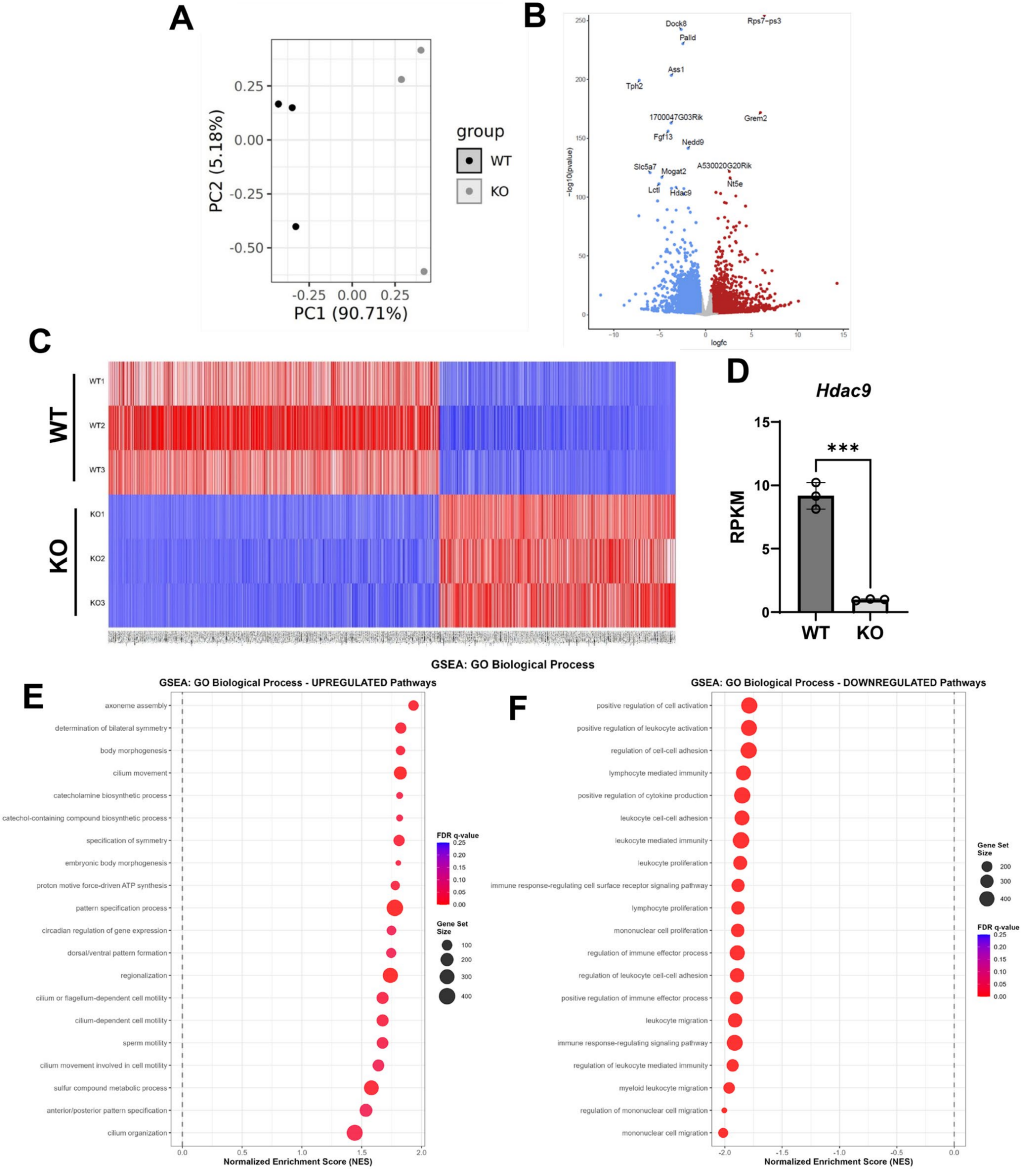
Differential gene expression between WT and HDAC9 KO mouse epididymal adipose tissue. (A) Principal component analysis (PCA) showing distinct separation in gene expression of VF from 10‐month‐old WT and KO mice. (B) Volcano plot where red dots represent significantly enriched or downregulated genes (adjusted < 0.01) in KO compared to WT VF. (C) Hierarchical clustering analysis heatmap of differentially expressed genes between WT and KO VF where red indicates higher expression and blue indicates lower expression. (D) RNA sequencing measurement of *Hdac9* expression in WT and KO VF. (E) Top 10 upregulated and (F) downregulated pathways identified by GSEA. VF, visceral adipose tissue; RPKM, reads per kilobase million; GSEA, gene set enrichment analysis.

To investigate the mechanisms whereby deletion of HDAC9 protects against adipose tissue senescence during aging, we performed RNA sequencing of visceral fat, specifically epididymal, from 10‐month‐old WT and *Hdac9* KO mice to evaluate gene expression profiles. Visceral fat was selected for this experiment because fat distribution shifts from the subcutaneous to the visceral compartment during aging, and this shift is associated with the development of age‐associated diseases [2]. The principal component analysis showed a distinct separation between the biological replicates for WT and *Hdac9* KO adipose tissues (Figure 4A). The dominant PC1 variance likely reflects biology, not technical variation. Base quality, RNA integrity, and batch conditions are uniform across all samples (Table S2), and the modest difference in read depth between groups (WT mean 56.0 M vs. KO mean 46.1M) is fully corrected by DESeq2's median‐of‐ratios normalization. The large PC1 variance is expected when comparing WT to complete knockout of class IIa histone deacetylase with broad chromatin‐remodeling activity as HDAC9 loss coordinately affects hundreds of gene loci, producing a dominant genome‐wide transcriptional signature. Subsequently, 2390 differentially expressed genes (DEGs) were identified, with 995 upregulated and 1395 downregulated in *Hdac9* KO relative to WT visceral fat (Figure 4B,C). This dataset also confirmed a significant reduction in *Hdac9* gene expression in *Hdac9* KO compared to WT visceral fat (Figure 4D). Gene set enrichment analysis (GSEA) identified signaling pathways that were significantly up‐ (Figure 4E) and down‐ (Figure 4F) regulated in *Hdac9* KO visceral fat. Consistent with our previous report that HDAC9 is a negative regulator of adipogenic differentiation (Chatterjee et al. 2011), RNA‐seq data also confirmed that genes associated with the adipogenesis pathway were significantly upregulated in *Hdac9* KO visceral fat (Figure 4E, Figure S3). Interestingly, metabolic pathways, including fatty acid metabolism, were among the top upregulated pathways in *Hdac9* KO compared to WT visceral fat (Figure 4E), and several pathways associated with inflammation, such as interferon gamma response, TNFα signaling via NFκβ, and IL‐6/JAK/STAT3 signaling, were significantly downregulated in *Hdac9* KO compared to WT visceral fat (Figure 4F). Additionally, the p53 pathway, which plays a central role in senescence, was the top downregulated pathway in *Hdac9* KO compared to WT visceral fat (Figure 4F), further suggesting an important role for HDAC9 in regulating adipose tissue senescence.

**FIGURE 5:**
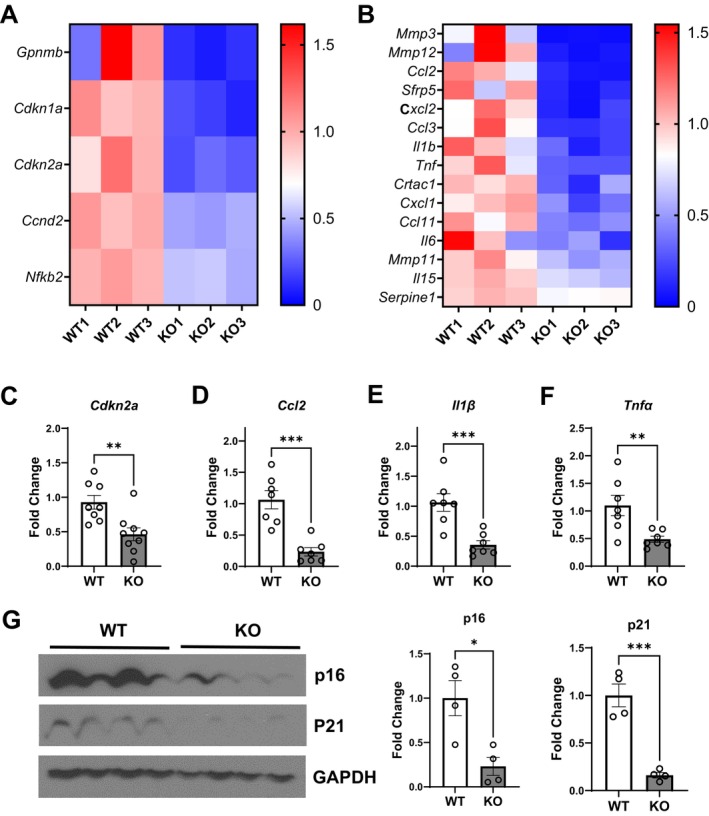
Reduced expression of senescence‐associated genes in visceral adipose tissue of *Hdac9* KO mice. Heat map of senescence (A) and SASP (B) gene expression from RNA sequencing of VF from 10‐month‐old WT and *Hdac9* KO mice (*n* = 3/group). (C‐F) qRT‐PCR validation of key senescence and SASP markers (C: Cdkn2a, D: Ccl2, E: Il1β, F: Tnfα, *n* = 7–9/group). (G) Representative Western blot and densitometry analysis of p16 and p21 expression in VF of 10‐month‐old WT and *Hdac9* KO mice (*n* = 4/group). SASP, senescence‐associated secretory phenotype. Data represent mean ± SEM. *p < 0.05, ***p < 0.001.

Expression of genes positively associated with senescence (Figure 5A) and the senescence‐associated secretory phenotype (SASP, Figure 5B) was reduced in visceral fat from *Hdac9* KO compared to WT mice. Reduced expression of representative senescence‐ (*Cdkn2a*) and SASP‐ (e.g., *Ccl2*, *Il1β*, *Tnfα*) associated genes was validated by qRT‐PCR (Figure 5C–F). Expression of P16 and P21 protein was also significantly reduced in visceral fat of *Hdac9* KO mice (Figure 5G). Furthermore, expression of genes associated with antioxidant activity (catalase) and peroxisomal biogenesis (*Pex3*, *Pex19*) trended to be higher in visceral fat of *Hdac9* KO mice (Figure S4).

### 3.5 HDAC9 Gene Deletion Increases Mitochondrial Content and Activity in Adipose Tissues

**FIGURE 6:**
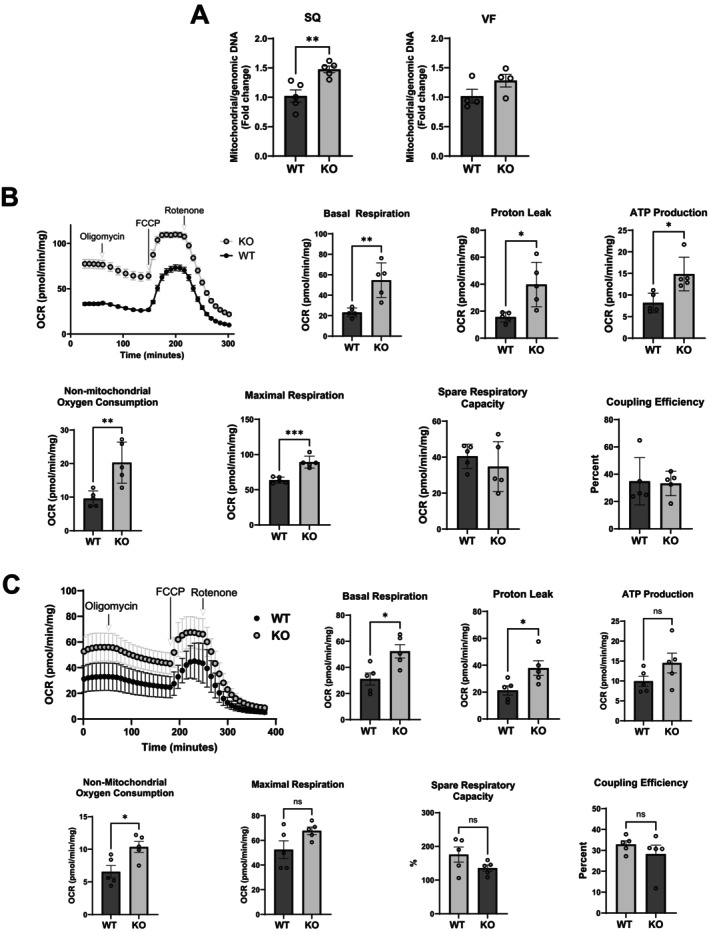
*Hdac9* gene deletion improves mitochondrial function in adipose tissues. (A) qPCR analysis of mitochondrial/genomic DNA content in adipose tissues from 10‐month‐old mice. Seahorse MitoStress assay of VF (B) and SQ (C) explants from WT and *Hdac9* KO mice (*n* = 5/group). Basal repiration, proton leak, ATP production, non‐mitochondrial oxygen consumption, maximal respiration, spare respiration capacity and coupling effiency were analyzed. The final concentrations of mitochondrial inhibitors were 10 μM oligomycin, 8 μM FCCP, 20 μM rotenone and 16 μM Antimycin A in Seahorse XF Dulbecco's DMEM. SQ, subcutaneous fat; VF, visceral fat. Data represent mean ± SEM. *p < 0.05, **p < 0.01.

Cellular senescence is mechanistically associated with impairment in mitochondrial function (Stab 2nd et al. 2016). Accordingly, we performed Seahorse analysis to assess mitochondrial function in subcutaneous adipose tissues from 3‐and 18‐month‐old mice. As expected, the 18‐month‐old mice weighed more than the 3‐month‐old mice, and we detected strong trends towards reduced basal respiration, proton leak, ATP production, and non‐mitochondrial respiration in adipose tissues of 18‐month‐old mice (Figure S5), suggesting impaired mitochondrial function. We previously reported that *Hdac9* KO mice had less fat/body mass and higher energy expenditure compared to WT mice despite similar food intake and locomotor activity (Chatterjee, Basford, Knoll, et al. 2014; Chatterjee, Basford, Yiew, et al. 2014). Thus, we hypothesized that the *Hdac9* KO mice might exhibit greater mitochondrial activity in adipose tissue compared to WT mice. Mitochondrial content, as measured by qRT‐PCR analysis, was increased in subcutaneous fat (Figure 6A, left panel) and trended higher in visceral fat (Figure 6A, right panel) from 10‐month‐old *Hdac9* KO mice compared to WT mice. Next, Seahorse MitoStress assay was performed using approximately 5 mg of freshly harvested adipose tissues from these mice. Interestingly, basal respiration and proton leak were higher in subcutaneous fat from *Hdac9* KO mice (Figure 6B), and trended higher in visceral fat from *Hdac9* KO mice (Figure 6C), as compared to WT mice, suggesting enhanced mitochondrial activity in adipose tissues of *Hdac9* KO mice. These results suggest that elevated mitochondrial content and activity may contribute to improved metabolic function and reduced senescence in *Hdac9* KO mice.

### 3.6 Thiosulfate Sulfurtransferase Is One of the Potential Downstream Mechanisms Regulated by HDAC9 in Adipose Tissues

**FIGURE 7:**
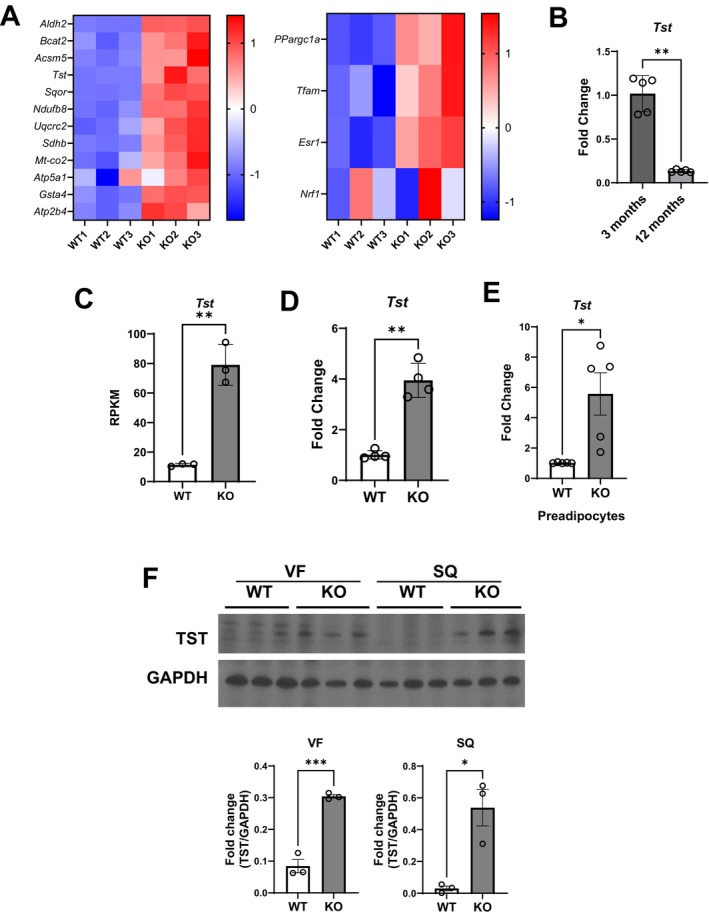
Reduced TST expression in adipose tissues and preadipocytes from Hdac9 KO mice. (A) RNA sequencing quantified relative expression of selected mitochondrial function (left panel) and mitochondrial biogenesis (right panel) genes in VF from 10‐month‐old WT and *Hdac9* KO mice (*n* = 3/group). (B) *Tst* gene expression from VF of 3‐ versus 12‐month‐old mice (*n* = 5/group). RNA sequencing quantification (C, *n* = 3/group) and qRT‐PCR validation (D, *n* = 4/group) of *Tst* in VF from WT and *Hdac9* KO mice. (E) *Tst* gene expression in preadipocytes isolated from WT and *Hdac9* KO mice was measured by qRT‐PCR (*n* = 5/group). (F) Western blot and densitometry analysis of TST protein expression in VF and SQ from 12‐month‐old WT and *Hdac9* KO adipose tissues (*n* = 3/group).

RNA‐sequencing also demonstrated upregulation of multiple genes associated with mitochondrial function and mitochondrial biogenesis in adipose tissues of *Hdac9* KO mice (Figure 7A, Figure S6). A previous report showed that higher TST expression in adipose tissues has been associated with leaner body weight in mice (Morton et al. 2016). TST also regulates levels of hydrogen sulfide, which plays an important role in cellular senescence (Kieronska‐Rudek et al. 2024). Interestingly, expression of *Tst* was significantly reduced in adipose tissues from 12‐month‐old compared to 3‐month‐old mice (Figure 7B), which was rescued by *Hdac9* gene deletion in 12‐month‐old mice as measured by RNA‐sequencing (Figure 7C) and qRT‐PCR (Figure 7D). Furthermore, *Tst* expression was also increased in preadipocytes isolated from *Hdac9* KO adipose tissue compared to WT (Figure 7E). Elevated TST protein expression in *Hdac9* KO mice was verified in visceral and subcutaneous fat from 12‐month‐old mice (Figure 7F).

**FIGURE 8:**
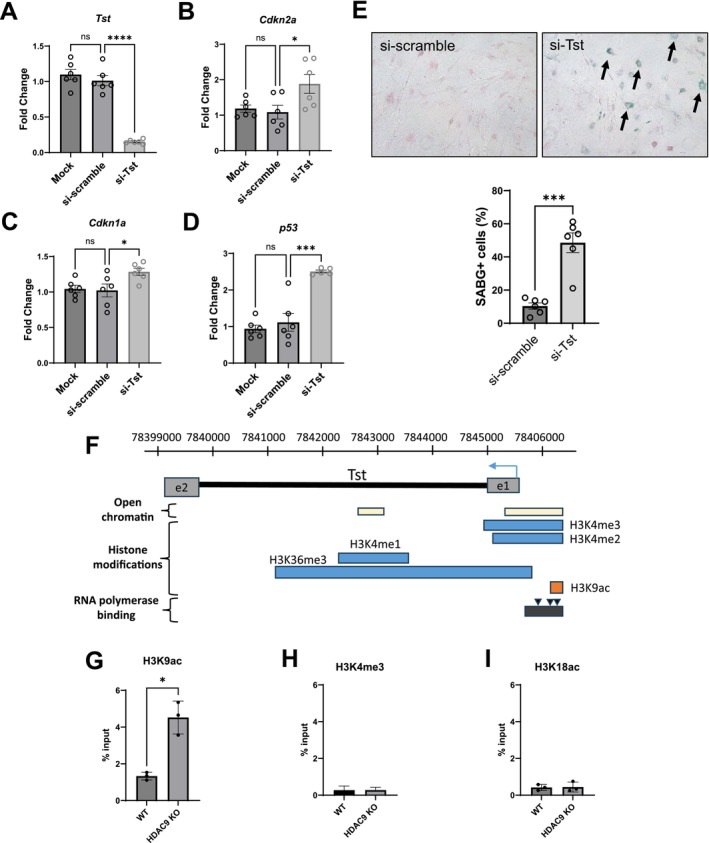
Tst is a potential mediator regulated by HDAC9 during preadipocyte aging. qRT‐PCR analysis of expression of *Tst* (A), Cdkn2a (B), Cdkn1a (C), and p53 (D) in preadipocytes from WT mice after si‐RNA knockdown of *Tst* (*n* = 6/group). Scrambled siRNA or non‐siRNA (mock transfection) groups were used as controls. (E) SABG‐staining (upper panel) and quantification (lower panel) in preadipocytes after si‐RNA knockdown of *Tst* (*n* = 6/group). Arrows indicate SABG positive cells. (F) Histone modification regions in *Tst* gene. Quantitative ChIP assays demonstrated increased levels of H3K9ac (G), but not H3K4me3 (H) or H3K18ac (I), at the promoters of *Tst* in HDAC9 KO adipose tissues. Data represent mean ± SEM. *p < 0.05, **p < 0.01, ***p < 0.001, ****p < 0.0001.

To investigate the functional role of TST in adipose senescence, *Tst* expression was silenced in preadipocytes using siRNA (Figure 8A–E). Interestingly, *Tst* knockdown significantly increased *Cdkn2a* (Figure 8B), *Cdkn1a* (Figure 8C) and *p53* expression (Figure 8D) in conjunction with increased number of SABG‐positive preadipocytes (Figure 8E), suggesting a potential association between reduced TST and adipose tissue senescence in aging mice. HDAC9 is known to regulate histone acetylation at H3K9 and H3K18 residues and public datasets indicate enrichment of H3K9 acetylation marks in the Tst promoter region (Figure 8F) (Beltram et al. 2016). To test whether HDAC9 directly regulates *Tst* transcription, we performed quantitative ChIP assays and observed increased H3K9 acetylation at the *Tst* promoter in Hdac9 KO preadipocytes (Figure 8G). In contrast, no significant changes were observed in H3K4me3 or H3K18ac (Figure 8H,I). These findings support a mechanistic link between HDAC9 activity and regulation of Tst transcription.

## 4 Discussion

Adipose tissue is a preeminent endocrine and energy storage organ that undergoes morphological, structural and functional changes during aging. However, the mechanisms responsible for these age‐related changes in adipose tissue are largely unknown. HDAC9 has previously been shown to regulate adipose tissue structure and function, and genetic deletion of *Hdac*9 protected against the development of diet‐induced obesity and insulin resistance in young mice (Chatterjee, Basford, Knoll, et al. 2014; Chatterjee, Basford, Yiew, et al. 2014). Furthermore, HDAC9 is associated with age‐related conditions, such as atherosclerosis, stroke and diabetes (Asare et al. 2025; Das and Natarajan 2020; Liu et al. 2024). Recently, we demonstrated that HDAC9 expression is downregulated in the aged brain and plays a role in age‐related cognitive decline and Alzheimer's disease (Lei et al. 2025). Here, we showed that *Hdac9* expression is elevated in adipose tissues of aging mice. *Hdac9* KO mice also exhibited reduced weight gain and adipose tissue senescence during aging. Mechanistically, *Hdac*9 gene deletion increased mitochondrial respiration in adipose tissues and increased expression of TST, a gene that has been associated with reduced senescence and increased leanness in mice (Morton et al. 2016). Finally, we demonstrated that knockdown of *Tst* in vitro promotes senescence in preadipocytes. Collectively, these findings suggest a novel role for HDAC9 in regulating adipose tissue aging, likely via modulation of expression of mitochondrial genes, including TST.

Aging was associated with increased expression of *Hdac9* in adipose tissues collected from mice, in conjunction with the accumulation of senescent cells. We previously demonstrated that HDAC9 expression is increased in adipose tissues in the context of obesity in mice fed a HFD (Chatterjee, Basford, Knoll, et al. 2014; Chatterjee, Basford, Yiew, et al. 2014). Furthermore, primary preadipocytes isolated from subcutaneous adipose tissue from mice fed a HFD exhibited impaired adipogenic differentiation, similar to that reported in aged mice (Kirkland et al. 1990). Increased senescence in adipose tissues has likewise been reported in mouse models of obesity (Wang et al. 2022). Impaired adipogenic differentiation and increased senescence of preadipocytes in aging and obesity may disrupt adipose tissue homeostasis due to mitochondrial dysfunction and inflammation converging on p21 and p16 pathways. Thus, further investigations are warranted to elucidate whether elevated expression of *Hdac9* can lead to a converging phenotype of adipose tissue dysfunction in the context of both obesity and aging.

Several recent studies have reported that senescent cells contribute to declining physical function during aging. Reducing senescent cell burden improves adipogenesis, insulin sensitivity, whole body energy expenditure, memory, and physical function in mice (de Oliveira Silva et al. 2025; Fang et al. 2025; Palmer et al. 2019; Xu, Palmer, et al. 2015; Xu et al. 2018). We found that *Hdac9* gene deletion reduced senescent cell burden in aging adipose tissues in vivo. Interestingly, *Hdac9* gene deletion also reduced cellular senescence induced by in vitro treatment of H_2_O_2_ or UV in isolated primary preadipocytes. These data suggest an intrinsic mechanism whereby reduced HDAC9 expression protects preadipocytes from becoming senescent, which in turn may preserve healthy adipose tissue function during aging.

To investigate the mechanisms whereby *Hdac9* gene deletion reduces adipose senescence during aging, we performed RNA sequencing and gene set enrichment analysis in visceral adipose tissues of middle‐aged mice. Consistent with our previous research on *Hdac9, s*everal pathways were upregulated in visceral fat by *Hdac9* gene deletion, including adipogenesis and fatty acid metabolism. In addition, we detected coordinated upregulation of genes associated with mitochondria biogenesis and function. The top downregulated pathways in adipose tissues of *Hdac9* KO mice included inflammatory signaling pathways and senescence itself. Importantly, mitochondrial function and inflammatory signaling are key regulators of cellular senescence. These data suggest that HDAC9 concomitantly regulates the expression of multiple genes/pathways and as such might function as a “master regulator” of senescence gene programs in adipose tissues.

To investigate the functional consequences of *Hdac9* gene deletion on mitochondrial activity and energy metabolism, we performed Seahorse analysis on freshly isolated adipose tissues. In WT mouse adipose tissues, aging reduced proton‐leak, ATP‐production and non‐mitochondrial respiration. These changes may contribute to the reduced energy expenditure and increased adiposity that occurs during aging (Manini 2010; Ou et al. 2022). Bioinformatics analysis of RNA sequencing data suggested that increased HDAC9 expression during aging could impair adipose tissue mitochondrial metabolism, a hypothesis that is supported by the Seahorse MitoStress data, which demonstrated that *Hdac9* gene deletion had the opposite effect of aging on parameters of mitochondrial respiration.

TST, also known as rhodanese, is a mitochondrial enzyme that was found to be upregulated in adipose tissues of *Hdac9* KO mice. TST catalyzes the transfer of a sulfur atom from a sulfur donor, such as thiosulfate, to a nucleophilic acceptor (Kruithof et al. 2020). TST was originally recognized as the enzyme responsible for cyanide detoxification; however, it has more recently been shown to have important roles in cellular function. TST regulates the cellular concentration of hydrogen sulfide (H_2_S), which is an important modulator of senescence and may play a role in adipose tissue function and diabetes (Kieronska‐Rudek et al. 2024; Zhu et al. 2020). TST is also involved in regulating iron–sulfur centers, such as those in complexes III and IV of the electron transport chain (Kruithof et al. 2020). TST is associated with sulfur transfer for the formation of glutathione persulfide, which is important for antioxidant defense (Kruithof et al. 2020). Additionally, TST has an important role in the generation of reactive selenium, leading to the formation of selenoproteins which have important roles in mitochondrial function (Kruithof et al. 2020). A recent study of a polygenic ‘lean’ mouse model identified TST expression as an important candidate gene that reduces adiposity (Morton et al. 2016). Overexpression of TST in adipocytes protected mice from diet‐induced obesity and insulin resistance and improved mitochondrial function (Acosta et al. 2013). Additionally, studies from human adipose tissues showed that TST expression is reduced in patients with obesity and type 2 diabetes compared to lean patients (Morton et al. 2016). Our studies further support the role of TST in regulating adipose function. TST expression was significantly reduced in adipose tissues of aged mice compared to young mice, and *Hdac9* gene deletion restored TST expression in adipose tissues of aged mice, suggesting that reduced TST expression may contribute to adipose tissue dysfunction during aging, at least in part as a consequence of elevated HDAC9 expression. Although previous study shows that HDAC9 has minimal catalytic activity during adipogenic differentiation (Chatterjee et al. 2011), ChIP data from this study suggests that HDAC9 enzymatic activity plays an important role in regulating *Tst* expression. Future studies using genome‐wide approaches such as ChIP‐seq will be necessary to comprehensively define the transcriptional mechanisms underlying *Tst* regulation. Furthermore, further studies investigating the functional contribution of TST in vivo will also be important to fully define its role in adipose tissue aging.

The present study has several limitations that are important to recognize. First, mechanisms of adipose tissue aging in different sexes are not fully understood. Similar to our previous studies using the global *Hdac9* KO mouse in the context of obesity (Chatterjee, Basford, Knoll, et al. 2014; Chatterjee, Basford, Yiew, et al. 2014), here we demonstrated that both male and female *Hdac9* KO mice have reduced body weight and adiposity in the context of aging. However, we previously demonstrated a sex difference in HDAC9 regulation of metabolism using an adipocyte‐specific *Hdac9* KO mouse, which was effective at protecting female, but not male, mice from diet‐induced obesity (Goo et al. 2022). Furthermore, differences in adiposity were less pronounced using the adipocyte‐specific KO model compared to that of the global KO model, suggesting that the adipocyte‐specific *Hdac9* gene deletion was not sufficient to fully protect male mice against the adverse effects of HFD feeding. Importantly, adipocyte‐specific *Hdac9* deletion did not significantly alter expression of senescence markers (p16, p21) in adipose tissue of male mice. These findings suggest that non‐adipocyte cell populations such as preadipocytes contribute to *Hdac9*‐mediated regulation of adipose tissue aging in male mice. Our previous work demonstrates that *Hdac9* expression is higher in the stromovascular fraction (SVF), particularly in male mice (Goo et al. 2022). Moreover, we also demonstrated that *Hdac9* expression in preadipocytes has been shown to impair adipogenic differentiation (Chatterjee et al. 2011), potentially limiting adipocyte turnover and contributing to adipose tissue aging. With aging, preadipocytes, the progenitor cells of adipose tissue, exhibit age‐related declines in proliferation and differentiation capacity that contribute to adipose tissue aging and dysfunction, and data obtained from preadipocyte models in this study also support this concept. Further in‐depth studies are required to fully define the mechanisms that regulate expression and function of HDAC9 in various cell types, and potential cell–cell crosstalk, within adipose tissues during aging. Second, adipose tissue heterogeneity can influence OCR measurements. Although we minimized variability by using the same anatomical depot, standardized explant size, and consistent normalization approaches, detailed histological assessment of fibrosis and stromal composition would further strengthen the conclusion. Our previous work demonstrated that Hdac9 deletion increases multilocular beige adipocytes in subcutaneous adipose tissue without significant differences in adipocyte size or inflammatory infiltration under chow diet conditions (Chatterjee, Basford, Knoll, et al. 2014; Chatterjee, Basford, Yiew, et al. 2014). Increased beige adipocyte abundance, which is associated with higher mitochondrial density, is consistent with the observed OCR phenotype, supporting the data of mitochondrial functions of adipose tissues in this study. Third, our previous study demonstrated that Hdac9 expression is influenced by environmental temperature and is increased under thermoneutral conditions (Ahmadieh et al. 2024). In this study, mice were housed at thermoneutrality to minimize variability related to cold‐induced thermogenesis. The relationship between temperature and adipose senescence remains incompletely understood. Finally, some experiments were conducted in 8‐ to 12‐month‐old mice, which are generally considered to correspond to middle age in humans; however, evaluation of metabolic phenotypes in mice aged 18–24 months would further strengthen conclusions regarding aging‐related effects. In addition, formal lifespan analysis was not performed because mice were sacrificed for tissue collection at predefined experimental time points. Future studies will be required to fully elucidate the mechanisms underlying HDAC9‐regulated adipose tissue aging.

In conclusion, our studies demonstrate a detrimental effect of elevated HDAC9 expression in adipose tissues during aging to promote adipose tissue senescence and mitochondrial dysfunction. Furthermore, reducing HDAC9 expression prevented senescent cell accumulation and improved metabolic activity of adipose tissues in aging mice. Therefore, HDAC9 may be a promising therapeutic target to improve healthy adipose tissue in aging.

## Author Contributions

B.G., S.A., H.W.K., N.L.W. conceived and designed research, B.G., S.A., P.V., H.S. D.S.K., M.O., R.C., N.C, S.C., L.L., Y.Z. performed experiments, B.G., S.A., P.V., H.S., L.L., Y.Z., H.W.K. analyzed data, B.G., S.A, H.W.K., N.L.W. interpreted results of experiments, B.G., H.W.K., N.L.W. wrote the manuscript, B.G., Q.D., H.W.K., Y.L., X‐.Y.L., N.L.W. edited and revised manuscript.

## Funding

This study was funded by grants NIH AG076235 (Xin‐Yun Lu and Neal L. Weintraub), AHA 971459 (Neal L. Weintraub), AHA 863622 (Neal L. Weintraub).

## Conflicts of Interest

The authors declare no conflicts of interest.

## Supporting information


**Figure S1:** acel70519‐sup‐0001‐DataS1.pdf.

## Data Availability

Datasets analyzed in this study are available from the corresponding authors upon reasonable request.
