# Supplementary material for: Histone Deacetylase 9 Gene Deletion Ameliorates Aging‐Related Adipose Tissue Senescence and Mitochondrial Dysfunction in Mice
Source: Aging Cell. 2026 Apr 25;25(5):e70519. doi: 10.1111/acel.70519 (PMC13109657; doi:10.1111/acel.70519)
Supplement: Supplementary file 1 — Figure S1: acel70519‐sup‐0001‐DataS1.pdf. [file ACEL-25-e70519-s001.pdf]

Supplementary material

Figure S1

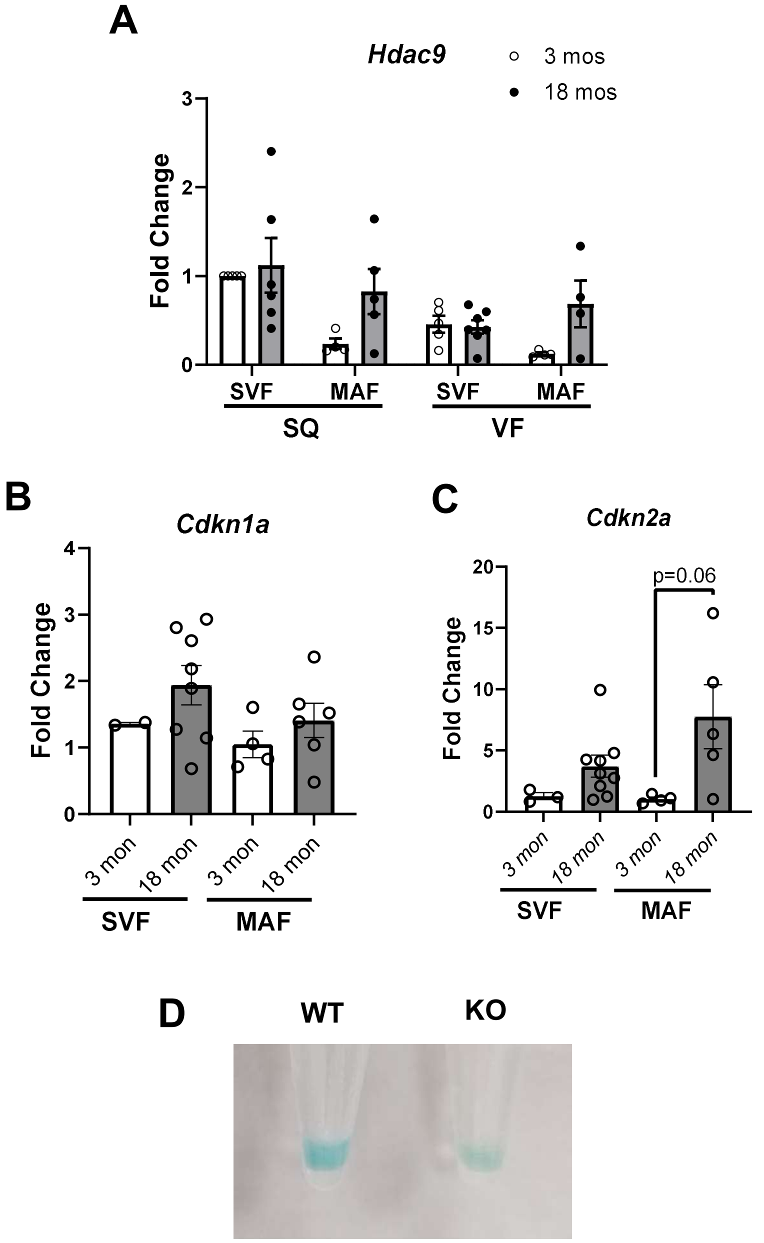

Figure S1: Effects of aging on *Hdac9* expression and senescence. (A) qRT-PCR analysis of *Hdac9* expression in SVF and MAF from SQ and VF from 3-month- or 18-month-old WT male mice (n=3-6/group). qRT-PCR analysis of *Cdkn1a* **(B)** and *Cdkn2a* **(C)** expression in VF from 3-month- or 18-month-old WT male mice (n=3-8/group). **(D)** SABG staining of MAF from VF of WT and *Hdac9* KO mouse adipose tissues.

Figure S2

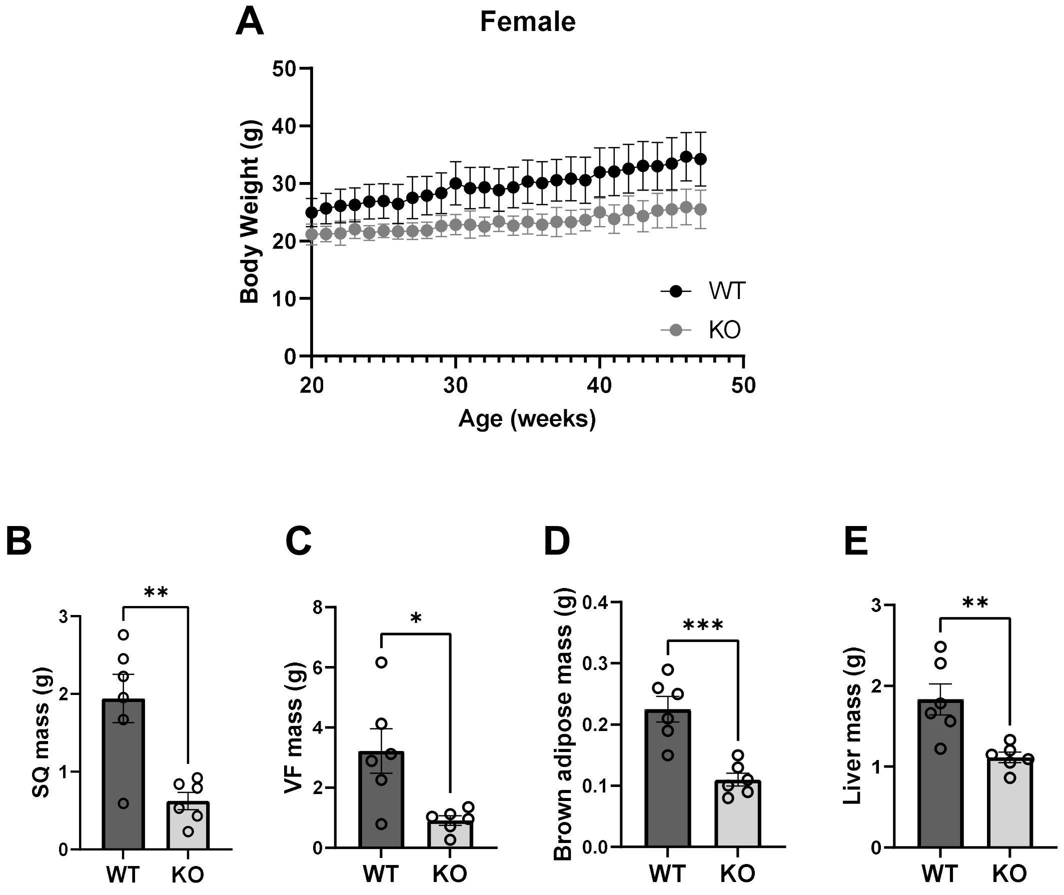

Figure S2: HDAC9 gene deletion leads to reduced adiposity in aging female mice. (A) Weekly body weight measurements of female WT and HDAC9 KO mice. Tissue mass of **(B)** SQ, **(C)** VF, **(D)** brown adipose, and **(E)** liver from 12-month-old female mice. n=6. Data represent mean ± SEM. *p<0.05, **p<0.01, ***p<0.001.

Figure S3

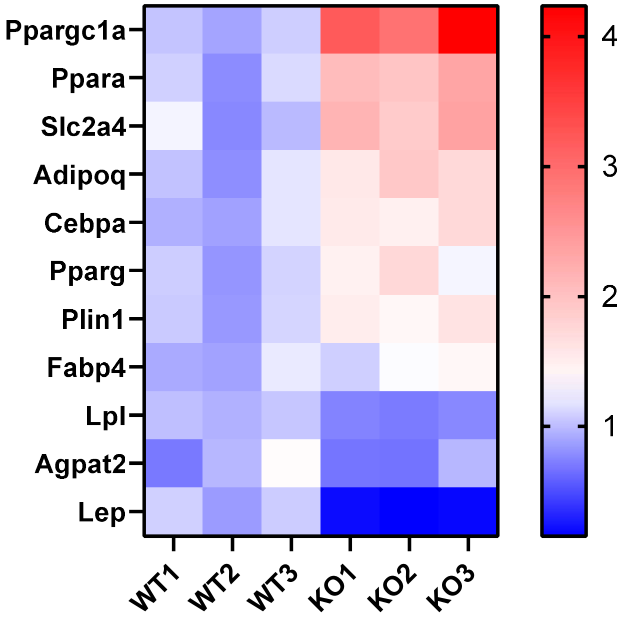

Figure S3: *Hdac9* gene deletion alters expression of adipogenesis-associated genes. (A) RNA sequencing quantified relative expression of selected adipogenesis-associated genes in VF from 10-month-old WT and KO mice. n=3.

Figure S4

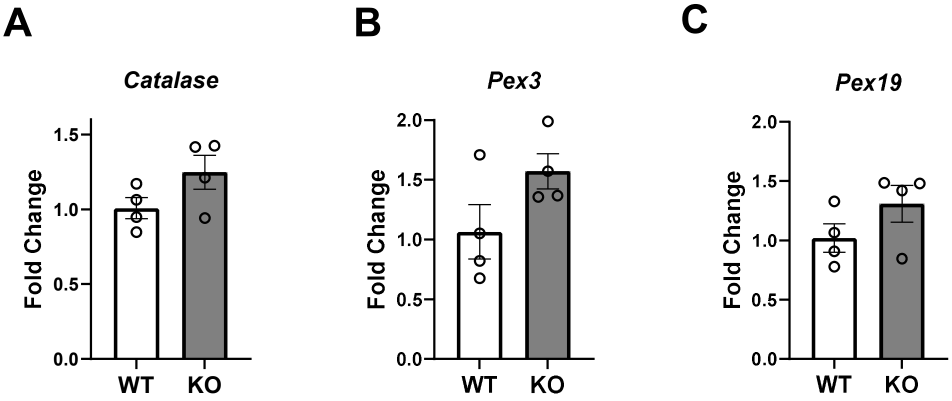

Figure S4: Effects of HDAC9 gene deletion on expression of peroxisome-associated genes. qRT-PCR analysis of expression of peroxisome-associated genes **(A)** *Catalase*, **(B)** *Pex3*, and **(C)** *Pex19* in VF of 10-month-old male mice. n=4.

Figure S5

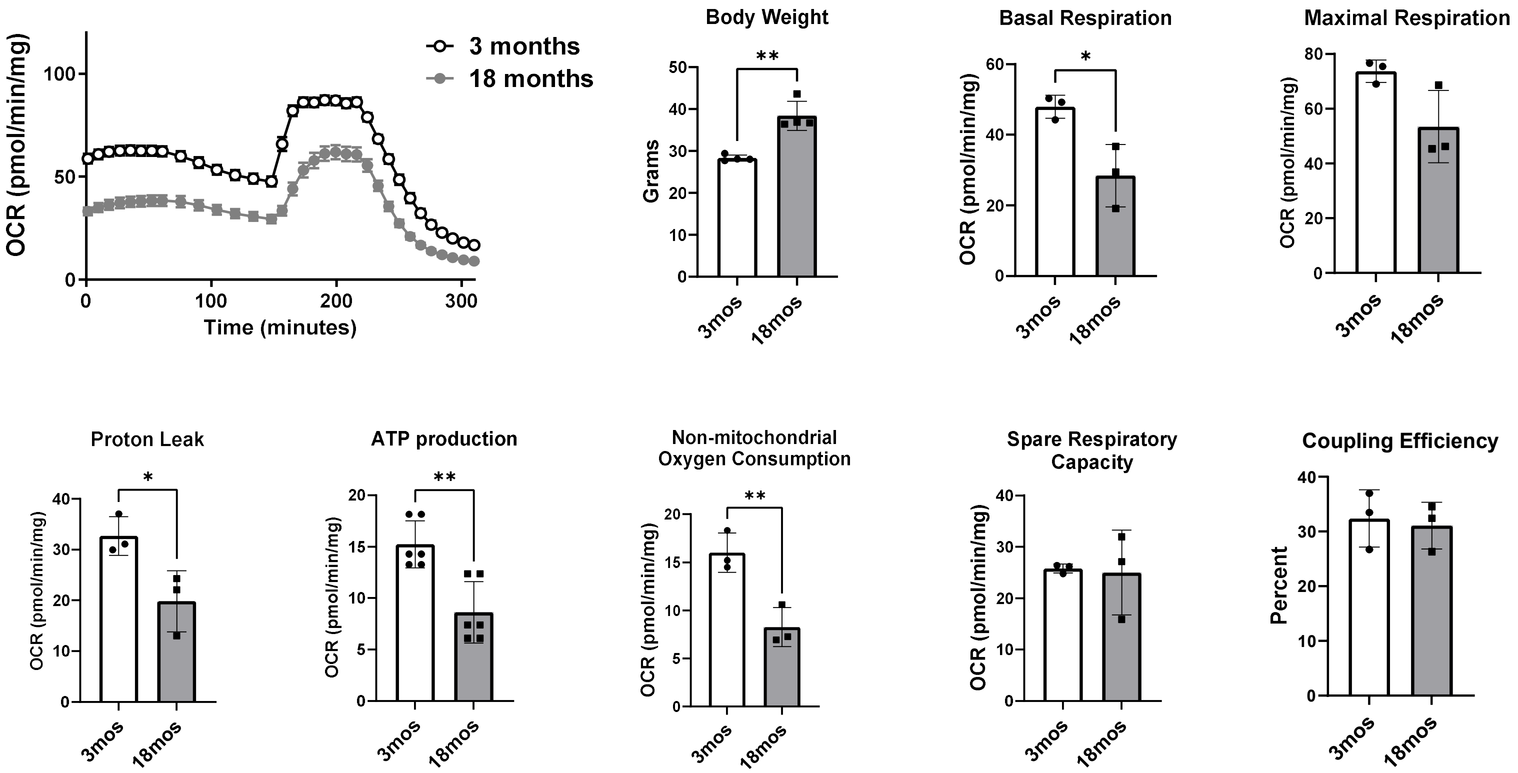

Figure S5: Mitochondrial function in subcutaneous adipose tissue of 3-month-old versus 18-month-old mice. Seahorse MitoStress assay of SQ explants from 3-month and 18-month-old WT mice. SQ, subcutaneous fat. Data represent mean ± SEM. *p<0.05, **p<0.01.

Figure S6

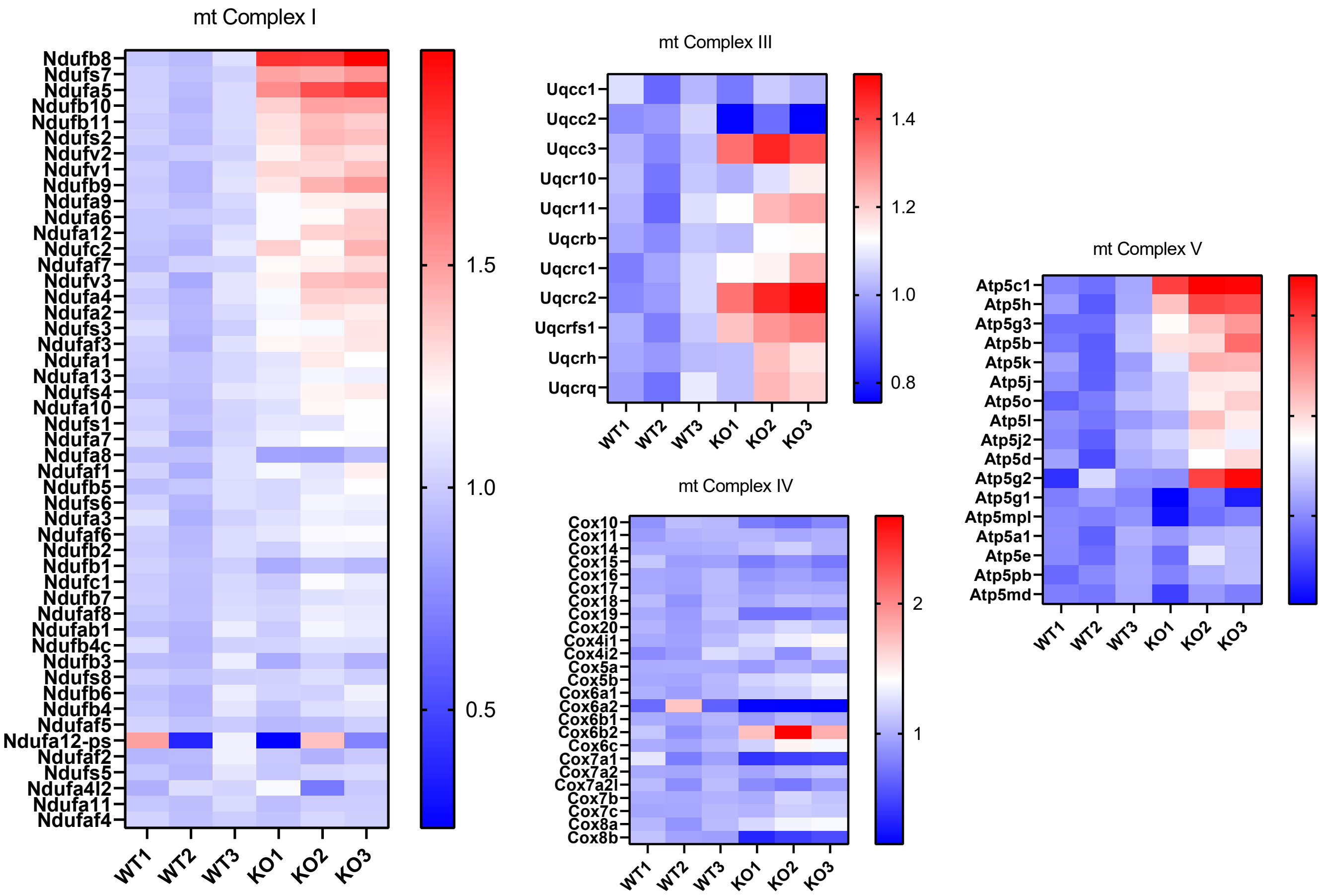

Figure S6: *Hdac9* gene deletion alters expression of genes associated with mitochondrial function. (A) RNA sequencing quantified relative expression of selected mitochondria-associated genes in VF from 10-month-old WT and KO mice. The heatmaps display Z-score normalized gene expression values calculated on a per-gene basis (row scaling) across samples. n=3

Figure S7

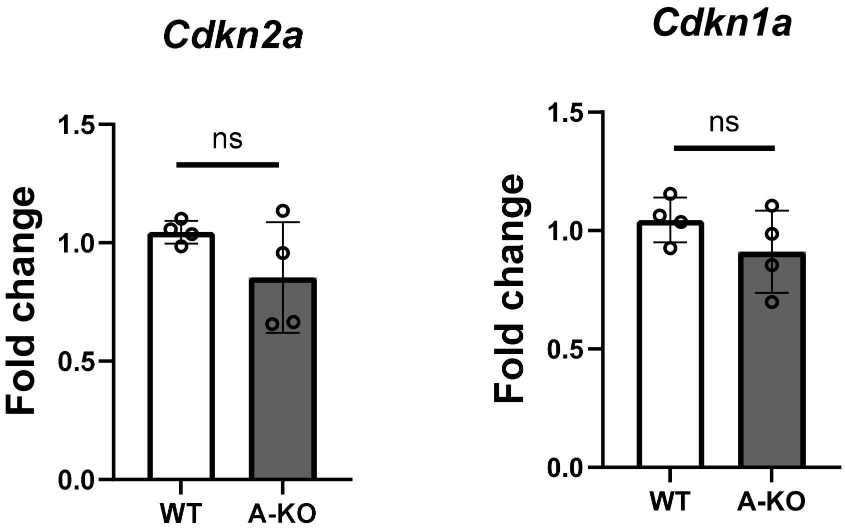

Figure S7: Adipocyte-specific *Hdac9* gene deletion did not alter expression of aging marker genes in SQ adipose tissues. Gene expression of Cdkn2a (p16, left panel) and Cdkn1a (p21, right panel) was measured in VF from 10-month-old adiponectin-cre positive (A-KO) and negative (WT) mice. n=4. Data represent mean ± SEM. ns = not significant.

Supplementary Table 1.

Primer sequences

Species	Gene	Forward primer	Reverse primer
Mouse	Adiponectin	GCACTGGCAAGTTCTACTGCAACA	AGAGAACGGCCTTGTCCTTGTTGA
Mouse	Arbp	AGCTGAAGCAAAGGAAGAGTCGGA	ACTTGGTTGCTTTGGCGGGATTAG
Mouse	B-globin	ATCCAGGTTACAAGGCAGCT	GGGAAACATAGACAGGGG
Mouse	Catalase	AAATGCTTCAGGGCCTT	GTAGGGACAGTTCACAGGTA
Mouse	Ccl2	GGCTCAAGCCAGATGCAGTTAC	GCCTACTCATTGGGATCATCTT
Mouse	Cdkn1a	CCTTGTCGCTGTCTTGCACTCT	GACCAATCTGCGCTTGGAGTGA
Mouse	Cdkn2a	CCCAACGCCCCGAACT	GAGCAGAAGAGCTGCTACGTGAA
Mouse	CoxII	CCATCCCAGGCCGACTAA	AATTTCAGAGCATTGGCCATAGA
Mouse	Hdac9	AGGATGATGATGCCTGTGGTGGAT	GAGTTGTGCTTGATGCTGCCTTGT
Mouse	Il1β	GCCGTCTTTCATTACACAGG	CTCCACCTCAATGGACAGAA
Mouse	Pex3	CCTAGGCAACCCACACAACA	TTGCAAGTCCTCTGGTTGCT
Mouse	Pex19	AGCATCATGCAGAACCTCCT	TGCTGCTGCTGGTACTTCTC
Mouse	Tnfα	CTTATCTACTCCCAGGTTCTCTTCAA	GAGACTCCTCCCAGGTACATGG
Mouse	Tp53	CAATGGAGGAGTCACAGTCG	GCAGAGGCAGTCAGTCTGAGTC
Mouse	Tst	CCTGCTGTAGGTTCACCTTTTAGG	GGAGGCACCAAGAGCAATTCTAAA

Supplementary Table 2.

RNA integrity metrics for RNA-seq samples
RIN and DV200 values for all six samples.

Sample ID	Sample Type	RIN for RNA	DV200 (for RNA)
**WT1**	Total RNA	9.3	90.22
**WT2**	Total RNA	9.7	91.59
**WT3**	Total RNA	9.1	91.2
**HDAC9 gKO1**	Total RNA	9.1	90.4
**HDAC9 gKO2**	Total RNA	8.9	89.44
**HDAC9 gKO3**	Total RNA	6.9	80.31

Sequencing metrics for RNA-seq samples
Read counts, yield, mean quality scores, and % bases ≥Q30 for all six RNA-seq libraries.

Sample ID	Barcode Sequence	# Reads	Yield (Mbases)	Mean Quality Score	% Bases >= 30
**WT1**	AAGCGTTC+ATAGAGAG	48926502	14678	37.96	90.88
**WT2**	AAGCGTTC+AGAGGATA	58093026	17428	38.04	91.16
**WT3**	AAGCGTTC+CTCCTTAC	61099492	18330	38.01	91.08
**HDAC9 gKO1**	AAGCGTTC+TATGCAGT	52859552	15858	37.96	90.87
**HDAC9 gKO2**	AAGCGTTC+TACTCCTT	43431803	13030	38.03	91.12
**HDAC9 gKO3**	AAGCGTTC+AGGCTTAG	42110544	12633	37.97	90.92
